# Extending the time window of mammalian heart regeneration by thymosin beta 4

**DOI:** 10.1111/jcmm.12421

**Published:** 2014-10-06

**Authors:** Liu Rui, Nie Yu, Lian Hong, He Feng, Han Chunyong, Meng Jian, Zheng Zhe, Hu Shengshou

**Affiliations:** State Key Laboratory of Cardiovascular Disease, Fuwai Hospital, National Center for Cardiovascular Diseases, Chinese Academy of Medical Sciences and Peking Union Medical CollegeBeijing, China

**Keywords:** neonatal mouse, EPDCs, cardiac regeneration, Tβ4

## Abstract

Recent studies demonstrated that the heart of 1-day-old neonatal mice could regenerate, with Wt1^+^ EPDCs migrating into myocardial regions after partial surgical resection, but this capacity was lost by 7 days of age. By treatment with Tβ4 to maintain Wt1 expression and retain the migrating feature of EPDCs in neonatal mice, we explored the possibility of restoring the cardiac regeneration potential of mice. We intraperitoneally injected Tβ4 into 1-day-old mice on daily basis and then apical resection was performed on the mice 7 days later. Twenty one days after the resection, morphological analysis revealed that the Tβ4-treated mice regenerated the resected ventricular apex, while the mice in PBS control group developed significant fibrosis without apical regeneration. The Tβ4-treated mice had significantly better ventricular ejection fraction and fractional shortening than controls. During the process of regeneration, Wt1^+^ EPDCs migrated into myocardial region and some of them expressed Islet1 and the markers for mature cardiomyocytes, such as cTnT and SαA. These characteristics of Wt1^+^ EPDCs were also seen in the heart regeneration of mice subjected to apical resection 1 day after birth. Tβ4 has no essential effect on cell cycle activity as no disruption of actin filaments was observed in Tβ4-treated hearts. These results revealed that the cardiac regeneration potential of neonatal mice could be extended to the 7th post-natal day by Tβ4 and Wt1^+^ EPDCs mobilization might play an important role in the extension.

## Introduction

The lost cardiomyocytes because of myocardial infarction are not replenished by new cardiomyocytes but by fibrotic cells because the adult mammalian heart possesses a measurable capacity for cardiomyocyte proliferation [1,2]. Albeit Porrello *et al*. exhibited that the hearts of 1-day-old mice could regenerate after partial surgical resection, this capacity was lost by 7 days of age [3]. It is unclear whether the time window of mammalian heart regeneration can be extended.

Recent research effort for prolonging heart regeneration capability has been directed at the epicardium, the epithelial cell sheet covering the heart. A number of studies reported that the epicardium play an important role in heart regeneration. In the epicardium, there are not only cardiac stem-cell niches but also telocytes, a particular type of interstitial cells [4–6], which form an interstitial network for stem cells [7] and cardiomyocytes [8]. After heart injury, telocytes can rebuild the network to regenerate the functional cardiomyocytes and to allow for angiogenese [8,9]. Moreover, foetal epicardium-derived cells (EPDCs), marked by Wilm's tumour 1 (Wt1), a foetal epicardial transcription factor launching epithelial-to-mesenchymal transition (EMT) in epicardium *via* triggering snai1 expression and indirectly inhibiting the expression of E-cadherin [10], can migrate into myocardial areas and differentiate into cardiomyocytes [11]. In adult mice, Wt1 is expressed at low levels and EPDCs are incapable of migration [12]. But, in 1-day-old mice, during heart regeneration, Wt1^+^ EPDCs were observed to migrate into myocardial area [3]. These studies collectively suggested that EPDCs of neonatal mice retain some capabilities of their foetal counterparts. We were led to hypothesize that maintaining Wt1 expression and epicardial traits of neonatal mice by treatment with thymosin beta 4 (Tβ4), which was shown to play a part in cell migration [13], re-expression of Wt1 in adult mice [14] and angiogenesis [15], can extend the cardiac regeneration capability of 1-day-old mice to the 7th day.

In this study, by treating neonatal mice with Tβ4, the hearts of 7-day-old mice were induced to regenerate the lost apex after apical resection. Moreover, the Wt1^+^ EPDCs were found to migrate into myocardial areas and to express the maturity markers of cardiomyocytes, such as cardiac troponin T (cTnT) and sarcomeric alpha actinin (SαA), at injury region.

## Materials and Methods

### Mice

We used CD-1 strain mice in the experiment and all experiments with mice were performed in strict accordance to the guidelines of and were approved by the Institutional Care and Use Committees of Fuwai Hospital or Peking Union Medical College. There were all 600 1-day-aged neonatal mice, which were divided averagely into four groups including operated group, sham-operated groups, Tβ4-treated group and PBS-treated group.

### Apical resection

Apical resection was performed on 1-day-old and 7-day-old mice by following the protocols previously reported by Porrello *et al*. [3]. About 90% of the 1-day-old neonates survived the surgical procedure. Although a smaller proportion of the apex was resected in 7-day-old pups, only 60% of them survived the operation. Sham-operated mice received the same procedure except for apical resection. Hearts were harvested 2, 4, 7, 14 and 21 days post-resection (dpr).

### Tβ4 administration

**Fig. 1:**
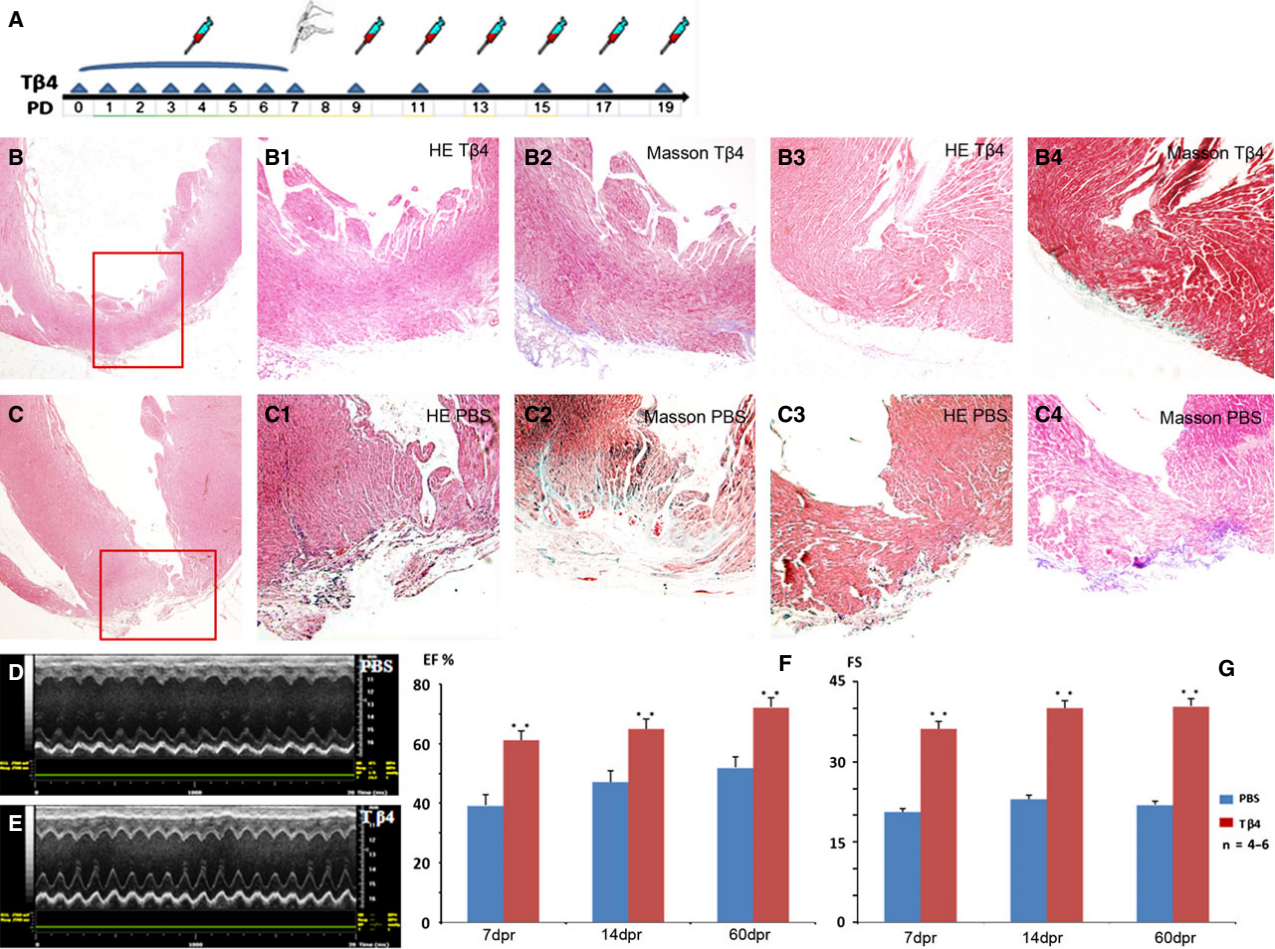
Restoration of heart regeneration potential of 7-day-old mouse. Schematic illustration of Tβ4 treatment (**A**). Haematoxylin and eosin staining of the mouse heart at 21 dpr showed in Tβ4-treated mice, the lost ventricular apex was regenerated, but in PBS group, the apex was still lost (**B**, **B-1**, **B-3**, **C**, **C-1**, **C-3**). Masson staining of serial sections showing minimal cardiac fibrosis (blue staining) in regenerated area at 21 dpr in Tβ4-treated mice (**B-2**, **B-4**), while in PBS group, obvious cardiac fibrosis could be found in injury region (**C-2**, **C-4**). Echocardiographically measured LV ejection fraction (EF%) and fractional shortening (FS) in both Tβ4 group and PBS group. The Tβ4-treated mice had significantly better ventricular ejection fraction and fractional shortening than controls.** mean *P* < 0.01 (**D**–**G**).

According to the reported protocol [14], the 1-day-old neonates were intraperitoneally injected with Tβ4 (Ray Biotech, Inc., Norcross, California, USA, at 12 mg/kg in PBS) or vehicle (PBS) for 7 days and apical resection was performed on these 7-day-old Tβ4- or PBS-primed mice. From the ninth day after birth, the mice received intraperitoneal injection of Tβ4 (Ray Biotech, Inc., at 12 mg/kg in PBS) or vehicle (PBS) on alternate days until the 19th day (Fig. 1A).

### Histological and immunohistochemical detection

Sections were stained with primary antibodies and secondary antibodies listed in Supplementary. Nuclei were counterstained by DAPI. Fluorescence was observed under a Leica: Wetzlar, Germany, SP8 confocal laser scanning microscope.

For quantification by ImageXpressMicro XL Widefield High Content Screening System, each group had 3–4 hearts and two slices were obtained from each heart. At each time-point, 6–8 slices of each group were scanned.

### Echocardiography

LV systolic function was echocardiographically assessed 7, 14 and 60 dpr after isoflurane anaesthesia by using a High-Resolution In Vivo Ultrasound Micro-Imaging System, equipped with a 30-MHz mouse ultrasound probe. There are 3–6 mice in each group at every time-point. Ejection fraction (EF) and fractional shortening (FS) was calculated on the basis of end-diastolic and end-systolic dimensions obtained by M-mode ultrasound.

### Statistical analysis

Data were expressed as the means ± SD and analysed by using SPSS 11.5 is the product of IBM SPSS company which lie in Chicago, Illinois, USA. Differences between groups were assessed by employing Student's *t*-test.

## Results

### Restoration of heart regeneration potential of 7-day-old mouse treated by Tβ4

Intraperitoneal injection of Tβ4 elevated the cardiac exogenous Tβ4 level in post-natal mice (Fig. S7). At 21 dpr, 10 Tβ4-treated and 10 PBS-treated mice heart samples were chosen randomly. And then morphological analysis revealed that in the Tβ4-treated mice, seven mice showed heart regeneration and the number was 0 in PBS-treated mice. In the Tβ4-treated mice, the lost ventricular apex regenerated with minimal fibrosis, but in PBS-treated group, the 7-day-old mice failed to regenerate their myocardium after apical resection and developed significant fibrosis (Fig. 1B and C; Fig. S1).

We echocardiographically assessed LV EF%, FS in Tβ4- and PBS-treated mice at 7, 14 and 60 dpr (Fig. 1D and E). In Tβ4 group, the EF% was 61.18 ± 7.92 at 7 dpr, 65.10 ± 7.51 at 14 dpr, 72.25 ± 6.26 at 60 dpr; the FS was 36.23 ± 0.98 at 7 dpr, 40.07 ± 5.95 at 14 dpr, 40.42 ± 5.48 at 60 dpr. In PBS (control) group, the EF% was 39.29 ± 2.72 (*P* = 0.00) at 7 dpr, 47.20 ± 1.25 (*P* = 0.00) at 14 dpr, 52.01 ± 4.03 (*P* = 0.00) at 60 dpr (Fig. 1F); the FS was 20.62 ± 3.73 (*P* = 0.00) at 7 dpr, 23.04 ± 0.74 (*P* = 0.00) at 14 dpr, 21.98 ± 4.04 (*P* = 0.00) at 60 dpr (Fig. 1G).

We also tested the EF% and FS in sham-operated group of both Tβ4-treated (STβ4-treated) and PBS-treated (SPBS-treated) mice at 7 and 14 dpr. Compared with Tβ4-treated, the EF% of STβ4-treated mice was 71.35 ± 4.49 (*P* = 0.04) at 7 dpr, 73.36 ± 4.90 (*P* = 0.06) at 14 dpr; the FS was 44.47 ± 2.05 (*P* = 0.00) at 7 dpr, 48.07 ± 4.01 (*P* = 0.06) at 14 dpr. Compared with PBS-treated, the EF% of SPBS-treated mice was 71.82 ± 4.99 (*P* = 0.00) at 7 dpr, 72.45 ± 4.37 (*P* = 0.00) at 14 dpr; the FS was 43.57 ± 2.15 (*P* = 0.00) at 7 dpr, 48.33 ± 4.33 (*P* = 0.00) at 14 dpr (Fig. S2).

### Migration and distribution of Wt1 marked EPDCs after heart injury in 7-day-old mice

**Fig. 2:**
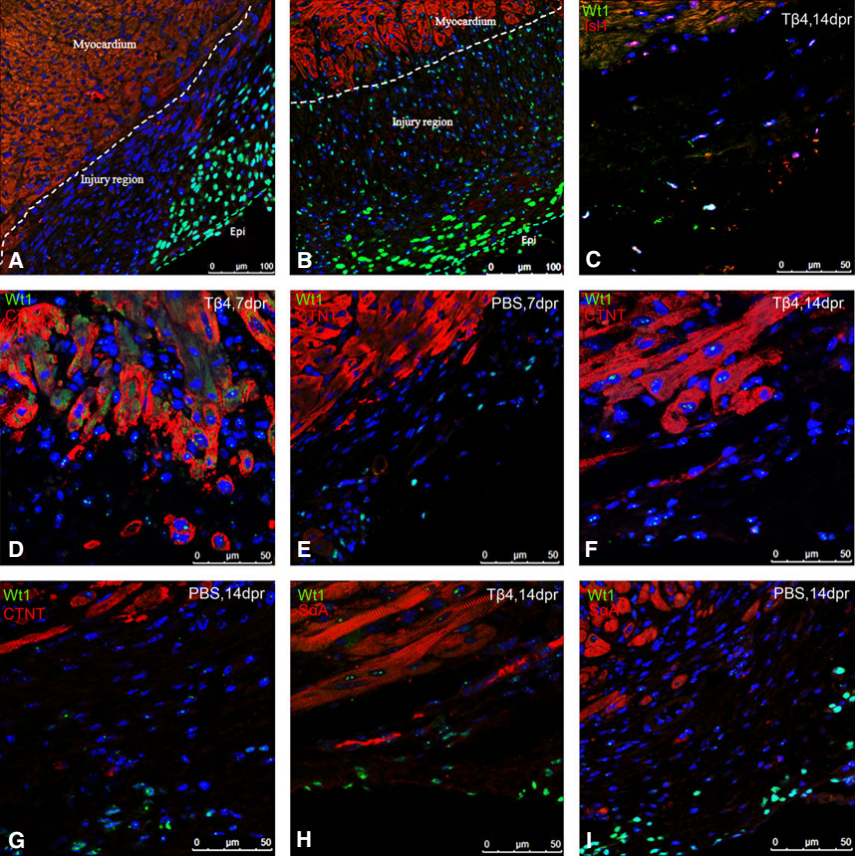
Migration of Wt1^+^EPDCs in the heart regeneration process of 7-day-old mice. Immunofluorescent staining showed Wt1^+^ EPDCs in PBS-treated hearts only stayed on the surface of the heart (**A**), Wt1^+^ EPDCs in Tβ4-treated hearts migrated into sub-epicardial and myocardial areas (**B**). Isl1 was expressed in Wt1^+^ EPDCs and these Wt1^+^/Isl1^+^ EPDCs were significantly increased in the regions of regenerated epicardium and sub-epicardium covering the resection site and across the entire injury area (**C**). At 7 dpr and 14 dpr, Wt1^+^ EPDCs in Tβ4-treated mice were located within the injury region and expressed cTnT (**D** and **F**) and SαA (**H**). In PBS-treated mice, no Wt1^+^/cTnT^+^ or Wt1^+^/SaA^+^ cells were observed (**E**, **G** and **I**). White dashed lines represent the border between myocardial and sub-epicardial areas; green dashed line indicates the border between epicardial and sub-epicardial areas.

By immunohistochemically staining Wt1, we established the Wt1-expression pattern at 7 and 14 dpr. After resection, in both groups, Wt1 expression was noted in epicardium, and epicardial cells increased from one layer to several layers. In Tβ4 group, Wt1^+^ EPDCs migrated into sub-epicardial and myocardial areas (Fig. 2B). At 7 dpr, Isl1, a marker of post-natal cardioblasts [16,17], was expressed in Wt1^+^ EPDCs (Fig. S3B and C) and at 14 dpr, these Wt1^+^/Isl1^+^ EPDCs were significantly increased in the regions of regenerated epicardium and sub-epicardium covering the resection site and across the entire injury area (Fig. 2C). But in PBS-treated mice, no Isl1 was expressed (Fig. S3E–G). At 7 and 14 dpr, some Wt1^+^ EPDCs in the injury area expressed the markers of cTnT (Fig. 2D and F) and SaA (Fig. 2H). On the other hand, in PBS group, the Wt1^+^ EPDCs stayed on the surface of the heart and did not migrate to myocardial areas (Fig. 2A). We failed to detect the expression of cTnT (Fig. 2E and G) or SaA (Fig. 2I) in Wt1^+^ EPDCs from 7 to 14 dpr.

### Generation and migration of Wt1-positive EPDCs after apical resection in 1-day-old mice

**Fig. 3:**
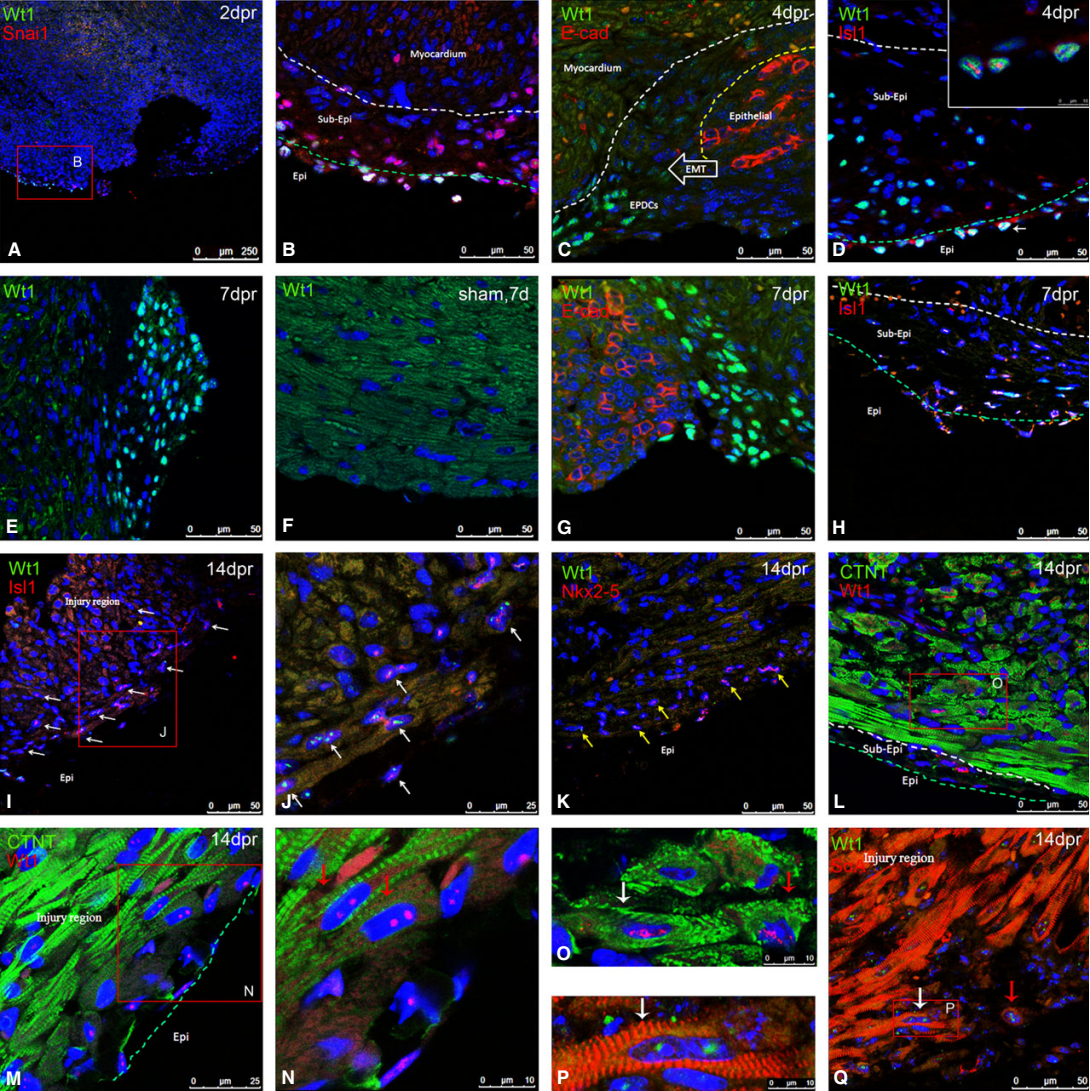
Generation and migration of Wt1^+^ EPDCs in 1-old-mice hearts. By immunostaining, snai1 was expressed in the Wt1^+^ epicardial cells at 2 dpr (**A** and **B**). No E-cadherin expression was observed in Wt1^+^EPDCs. In the Wt1^−^ epicardial cells, E-cadherin was expressed around the cells (**C** and **G**). Epicardial thickening and Wt1 up-regulation were most pronounced in the vicinity of injury area (**E**), while only few Wt1^+^ cells were found in the sham-operated heart (**F**). Wt1^+^/Isl1^+^ cells stayed in the epicardial area adjacent to injury regions at 4 dpr (**D**) and increased at both epicardial and sub-epicardial layers covering injury region at 7 dpr (**H**). At 14 dpr, white arrows indicated that these double-positive cells were located throughout the injury area (**I** and **J**). Yellow arrows showed Nkx2-5^+^/Wt1^+^ cells lay in the injury area at 14 dpr (**K**). Wt1^+^/cTNT^+^ and Wt1^+^/SαA^+^ cells were seen in myocardial area adjacent to injury region (**L**, **M** and **Q**). White arrows indicated Wt1^+^/cTNT^+^ and Wt1^+^/SαA^+^ cells which were filled with myofibrils with repeated bands (**O** and **P**) and Red arrows showed these cells with peripherally located (**N**) or irregularly arranged (**Q**) myofibrils. White dashed line indicates the border between myocardial and sub-epicardial areas; green dashed line represents the border between epicardial and sub-epicardial regions.

We re-performed apical resection in the 1-day-old neonate mice (Fig. S4 A1–A4). After the partial ventricular amputation, Wt1 was strongly expressed (Fig. S4B). Epicardial cells increased from one layer to several layers especially in the vicinity of injury area (Fig. 3E, Fig. S4G and I), covering the ventricles, atria and out-flow tract within the first 7 days (Fig. S4C–F), enveloping the wound and migrating into sub-epicardial and myocardial regions (Fig. S4K). A marginal level of Wt1 expression was observed in epicardial cells in sham-operated mice (Fig. 3F, Fig. S4H and G). Ki67 staining exhibited that most Wt1^+^ cells were in mitosis stage (Fig. S4L). At 21 dpr, when the resected heart completely regenerated, only few Wt1^+^ cell could be observed (Fig. S4I). Quantification of Wt1^+^ cells by high-content microscopy (HCS) showed that the percentage of Wt1^+^ cells in the entire heart was significantly higher than that in the sham-operated hearts at 2, 7 and 14 dpr (*n* = 3–6 each group, mean ± SD; Fig. S4M, Table S1).

To examine EMT of epicardial cells, we immunohistochemically detected the expression of snai1 and E-cadherin in injury region. Concomitant with the migration of EPDCs to sub-epicardial area, expression of snai1 was found to be in the Wt1^+^ epicardial cells at 2 and 4 dpr (Fig. 3A and B; Fig. S5A and B). No snai1 was observed in sham-operated mice heart (Fig. S5E–H). E-cadherin protein expression was suppressed in Wt1^+^ EPDCs, while the protein surrounded the Wt1^−^ epithelial cells of epicardium (Fig. 3C and G).

To examine the migration of Wt1^+^ EPDCs, we conducted a systematic longitudinal study by immunohistochemically staining Wt1 after heart resection. Some Wt1^+^ EPDCs were found to express Isl1 at 4 dpr (Fig. 3D). These Wt1^+^/Isl1^+^ EPDCs were significantly increased at both epicardial and sub-epicardial areas covering injury region at 7 dpr (Fig. 3H). By 14 dpr, the cells were distributed across the whole injury area (Fig. 3I and J). And Nkx2-5, an early marker of cardiomyocyte progenitors [18,19], was expressed in some of Wt1^+^ EPDCs at the injury area at 14 dpr (Fig. 3K). At 14 dpr, some Wt1^+^ EPDCs were found to be within the injury region and expressed both cTnT (Fig. 3L–O) and SaA (Fig. 3P and Q). Quantification of Wt1^+^ cells by HCS showed that the percentage of Wt1^+^/cTnT^+^ cells in the entire heart were higher than that in the sham-operated hearts at 4, 7 and 14 dpr (Fig. S6, Table S2).

### Tβ4 has no essential effect on cell cycle activity

**Fig. 4:**
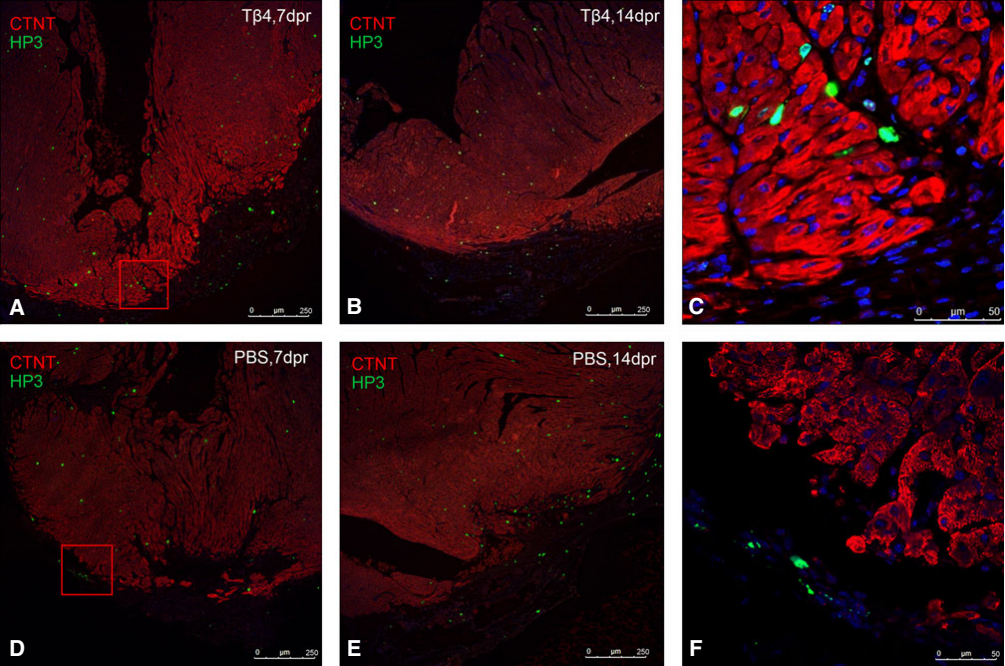
Tβ4 has no essential effect on cardiomyocyte self-proliferation. PH3^+^ cells were stained in PBS- and Tβ4-treated heart slices at 7 (**A** and **D**) and 14 dpr (**B** and **E**). Red box indicated most of PH3^+^ cells lay in nucleus of interstitial cells (**A**) and cells in epicardial region (**D**). The high-magnification image of which were **C** and **F** respectively. The cardiomyocytes were filled with regularly arranged myofibrils with repeated bands (**C** and **F**).

To explore whether Tβ4 slows the cell cycle withdrawal process in Tβ4-treated hearts by disrupting actin filaments in cardiomyocytes, cardiomyocyte mitosis was assessed by co-localization of phospho-histone H3 (PH3) and cTnT in PBS- and Tβ4-treated hearts at 7 and 14 dpr. A lot of PH3^+^ cells were stained in the heart slices of both groups (Fig. 4A, B, D and E). But most of PH3 was expressed in nucleus of interstitial cells (Fig. 4C) and some PH3^+^ cells were in epicardial region (Fig. 4F) in both groups. No obvious evidence of sarcomere disassembly, marginalization of sarcomeric structures to the periphery of the cells, was observed in the two groups (Fig. 4C and F).

### Tβ4 improved angiogenesis in injury area

**Fig. 5:**
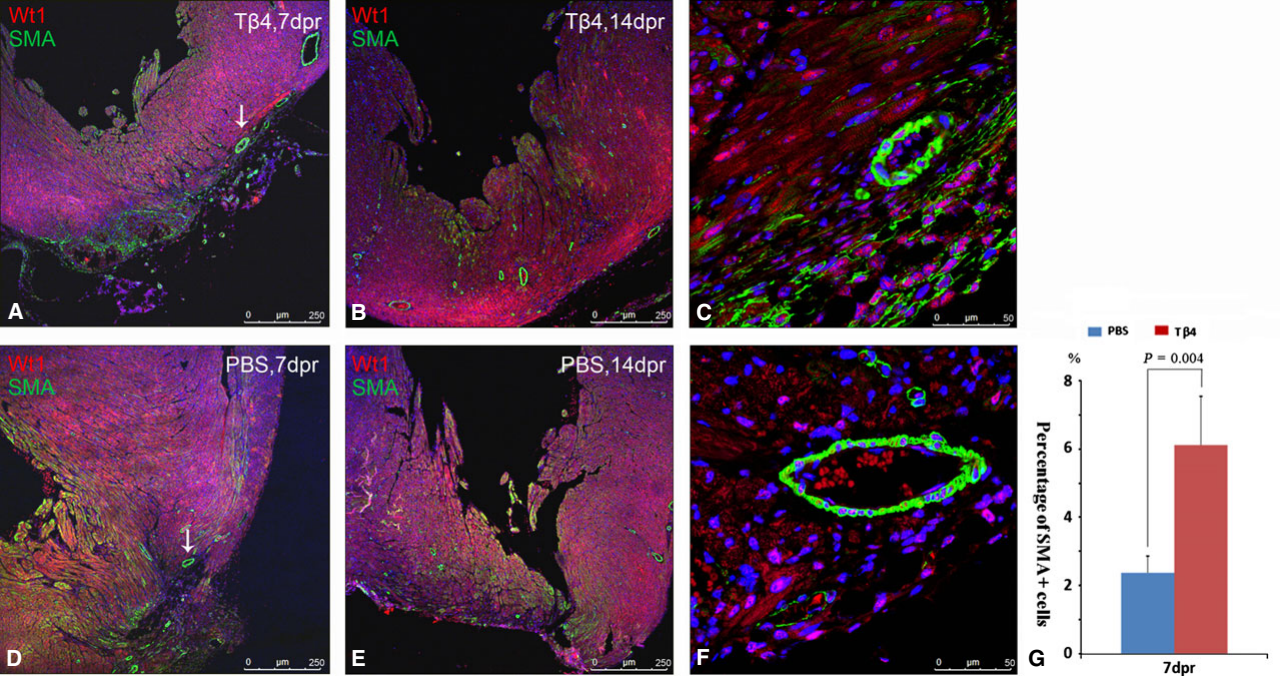
Tβ4 improved angiogenesis.SMA^+^ vessels were immunohistochemically visualized in PBS- and Tβ4-treated hearts at 7 (**A** and **D**) and 14 dpr (**B** and **E**). White arrow indicated the SMA^+^ vessels of injury region. Wt1^+^/SMA^+^ double-positive cells were in the wall of vessel in both groups (**C** and **F**). Quantification of SMA^+^ cells in two-third of the ventricle containing the regeneration plane by HCS showed that the percentage of the cells in Tβ4-treated hearts was significantly higher than that of PBS-treated group (**G**; *n* = 3 in each group, *P* = 0.0038).

To study the vascular promotion effect of Tβ4 in injury area, we stained smooth muscle actin (SMA) and Wt1 in PBS- and Tβ4-treated hearts at 7 and 14 dpr. SMA-positive vessels were immunohistochemically visualized in the heart slices of both groups at 7 and 14 dpr (Fig. 5A, B, D and E). Quantification of SMA^+^ cells by HCS showed that at 7 dpr, the percentage of SMA^+^ cells in two-third of the ventricle containing the regeneration plane was 6.13 ± 1.41 in Tβ4-treated hearts and for PBS-treated hearts, the percentage was 2.39 ± 0.49 (*P* = 0.0038; Fig. 5G). Moreover, in both group, the cells positive for both Wt1 and SMA were observed within coronary vessels in injury region and adjacent areas at 7 and 14 dpr (Fig. 5C and F).

## Discussion

In this study, we showed that 7-day-old mice treated by Tβ4 could restore heart regeneration potential. During the process of regeneration, the Wt1^+^ EPDCs increased dramatically and migrated into myocardial areas. Moreover, co-localization of Islet1, cTnT and SαA, respectively, with Wt1 was observed. All these EPDC features were observed in 1-day-old neonatal mice. In PBS-treated group, no similar phenomena were seen. These results suggested that treatment with Tβ4 could retain the traits of EPDCs in neonatal mice and the Wt1^+^ EPDCs contributed to the heart regeneration of 7-day-old mice.

In both Tβ4- and PBS-treated groups, PH3 was mostly expressed in nuclei of interstitial cells and the cells in epicardial region, and no obvious evidence of sarcomere disassembly was noted, suggesting that Tβ4 exerts no substantial effect on cardiomyocyte proliferation. The reason may be that, in the 7-day-old mice, cardiomyocyte proliferation was arrested in rodents. Although it was previously reported that Tβ4 enhanced cell migration [13] and improved reprogramming of murine fibroblasts to cardiomyocytes [20], no report showed that Tβ4 could prolong cell cycle. This study showed that Tβ4 could retain the EPDC features of neonatal mice, including migration and co-localization of Islet1, cTnT and SαA, respectively, with Wt1. So it is possible that Tβ4 promotes reprogramming of Wt^+^ EPDCs to cardiomyocytes during heart regeneration in 7-day-old mice.

According to recent reports [13], Tβ4 can induce adult EPDCs neovascularization after heart injury. In this study, in both Tβ4- and PBS-treated groups, the WT1/SMA-positive cells were within coronary vessels, indicating that Wt1^+^ EPDCs might adopt the fate of smooth muscle cells thereby contributing to angiogenesis. The increased SMA^+^ vessels in the Tβ4-treated group made a role in recovering of heart function.

The study still had some limitations. For immunohistochemical co-localization, in each group, we tested six hearts at each time-point and stained at least three slices from each heart. And that, we employed two kinds of secondary antibodies with different colours to stain one marker. But we noticed that in the epicardial region, Wt1 was expressed in whole nuclei staining pattern, while in myocardial region, Wt1 was expressed in nucleoli-like staining pattern. The reason might be the down-regulated expression of Wt1 in the myocardial area. Even so, the possibility of staining artefact could not be altogether eliminated. We were not entirely sure that Wt^+^ EPDCs were reprogrammed to cardiomyocytes. In our following studies, the fate of Wt^+^ EPDCs will be examined by using Wt1^GFPCre/+^ and Wt1^CreERT2/+^; R26R^EYFP/+^ mice. Moveover, the contribution of other circulating stem cells will be studied.

## Supporting information

**Figure S1** Regeneration potential of 7-day-old mouse. **Figure S2** Heart function and Masson staining at 7 dpr and 14 dpr. **Figure S3** Co-localization of Wt1 with Isl1 in the heart regeneration process of 7-day-old mice. **Figure S4** Activation of epicardium after apical resection in 1-day-old mice. **Figure S5** The expression pattern of snai1 in the heart regeneration process of 1-day-old mice. **Figure S6** Microscopy images and image reconstruction for Wt1 and cTNT staining in 1-day-old-mice heart. **Figure S7** Intraperitoneal injection of Tβ4 could elevate the cardiac exogenous Tβ4 level in heart of post-natal mice. **Table S1** The percentage of Wt1+ cells in whole heart in the heart regeneration process of 1-day-old-mice heart. **Table S2** The percentage of Wt1+/cTNT cells in whole heart in the heart regeneration process of 1-day-old-mice heart.
