# Supplementary material for: Extending the time window of mammalian heart regeneration by thymosin beta 4
Source: J Cell Mol Med. 2014 Oct 6;18(12):2417–24. doi: 10.1111/jcmm.12421 (PMC4302647; doi:10.1111/jcmm.12421)
Supplement: Supplementary file 1 [file jcmm0018-2417-sd1.docx]

Supplementary Material for

**Extending the time window of mammalian heart regeneration by thymosin beta 4**

***Western blotting***

Western blotting for Wt1 protein was performed on a NuPAGE electrophoresis system (Invitrogen). Primary antibodies were rabbit monoclonal anti-Wilms tumor antibody (1:3000, abcom, ab89901), and goat anti-rabbit antibody (1:20,000, abcom, ab136817) was used as secondary antibody. Bands were visualized by using an enhanced chemiluminescense (ECL) kit (GE Healthcare).

***Histological and Immunohistochemical detection***

The heart samples were fixed in 4% paraformaldehyde overnight and then paraffin- embedded. Paraffin-embedded sections (3 μm) were cut through the entire ventricles and atria. Hematoxylin/eosin (HE) and Masson’s trichrome staining were performed according to standard protocols. Slides were de-paraffinized, permeabilized with 0.3% Triton X-100 in PBS after antigen retrieval, blocked in 10% goat serum (Invitrogen) and incubated with primary antibody overnight at 4ºC. On the following day, slides were washed 3 times in PBS and then incubated with the second antibody for 45 min at 37°C. Slides were again washed 3 times in PBS in a dark room and then mounted with Dapi-Fluoromount-G™ (Southern Biotech). The primary antibodies included Wt1 (Rabbit monoclonal anti-Wilms tumor antibody, 1:250, abcom, ab89901), cTNT (Mouse monoclonal anti-cardiac Troponin T antibody, 1:500, abcom, ab8295), Islet1 (Mouse monoclonal anti-Islet1 antibody, 1:500, Santa Cruz, SC-101072), Nkx2-5 (Mouse monoclonal anti-Nkx2-5 antibody, 1:500, Santa Cruz, SC-376565), HP3(Rabbit monoclonal anti-phospho-histone H3 antibody, 1:250, abcom), SMA(Rabbit monoclonal anti- smooth muscle α-actin antibody, 1:250, abcom ), Snai1 (Mouse monoclonal to mouse, 1:1000,Cell Signaling, 3895), E-caderin (Mouse monoclonal to mouse, 1:500, abcom, ab76055), Sarcomeric alpha actinin antibody (Mouse monoclonal to mouse, 1:500, abcom, ab9465), Sarcomeric alpha actinin antibody (Rabbit polyclonal to mouse,1:500, abcom, ab137346), Tβ4 (Rabbit polyclonal to mouse,1:500, abcom). The secondary antibody included Alexa Fluor 488 (Donkey anti rabbit , 1:500, Invitrogen), Alexa Fluor 594 (Donkey anti mouse , 1:500, Invitrogen), FITC (Goat anti mouse, 1:500, Invitrogen) and Alexa Fluor 555 (Donkey anti rabbit , 1:500, Invitrogen). Fluorescence was observed under a Leica SP8 confocal laser scanning microscope and the number of cells were quantitatively determined on an ImageXpress Micro XL High-content Screening System (Molecular Devices).

***High-content microscopy***

High content screening (HCS) utilizes automated, high-resolution microscopy systems to assay and visualize phenotypic responses. Tissues or cells in slides or in wells are imaged at high speed, then morphological information is extracted from fluorescent or bright field images by robust analysis software. The complete high content imaging portfolio provide the ultimate flexibility and performance to perform tailored assays and shorten time to result. Robust imaging systems packaged with intelligent analysis software integrated data management and visual informatics.

Here, in order to test the percentage of the certain cells in entire heart. Using HCS we screened a series of heart slicing at 4, 7, 14 dpr in both sham and resected mice heart. There are 2 slicing through every heart and 3-4 mice heart in each group at every time point. Together 6-8 slides in each group were screened and imaged at high speed. The percentage of these cells of each slicing were analyzed by intelligent analysis software.

**
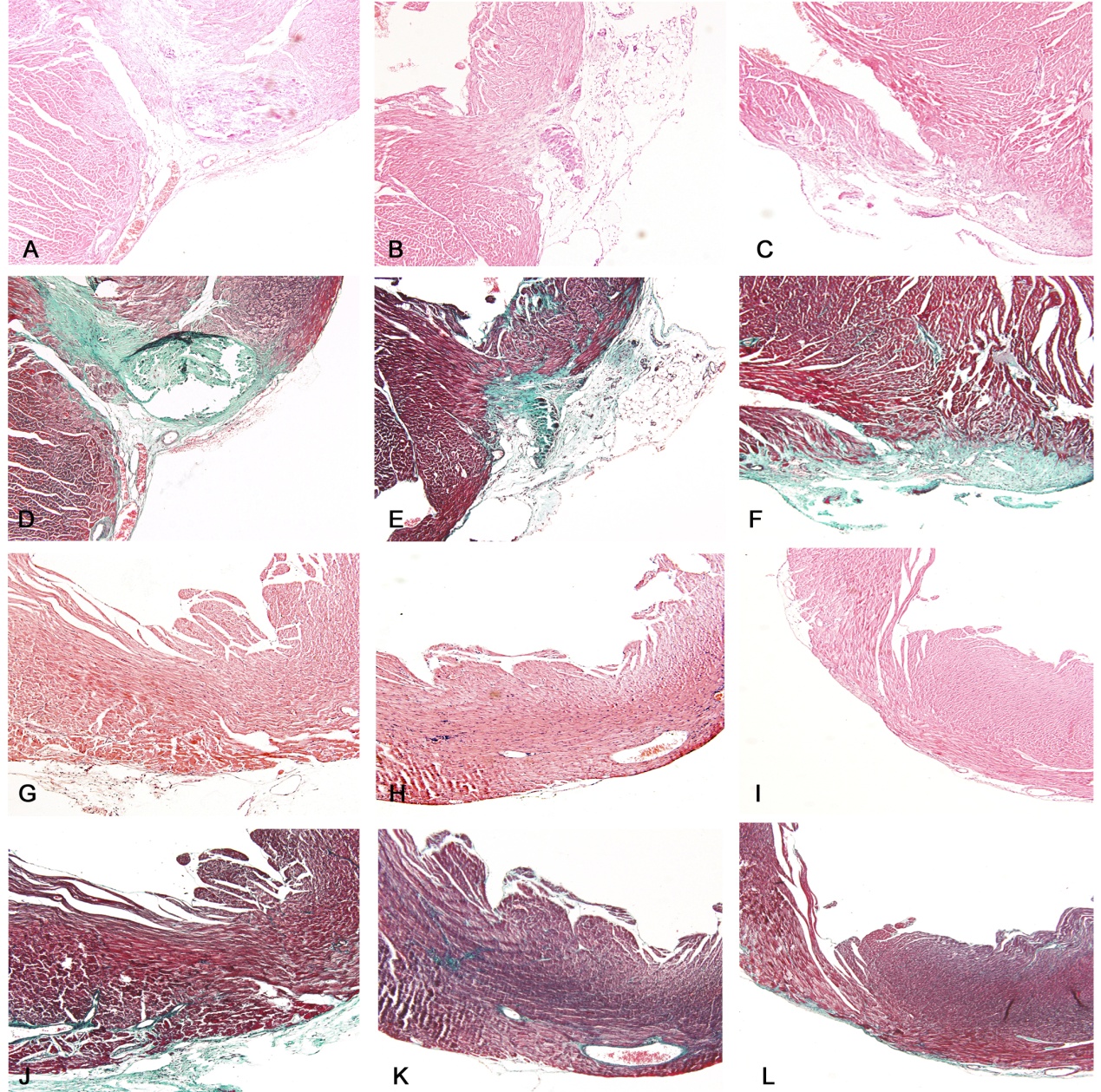
**

**Supplementary figure 1: Regeneration potential of seven-day-old mouse.** H&E staining showed in PBS-treated mice the apical myocardium was still lost (A-C) and masson-staining of serial sections showing obvious cardiac fibrosis could be found in injury region at 21 days post-resection (dpr) (D-F). In Tβ4-primed mice the lost ventricular apex was regenerated (G-I) with minimal cardiac fibrosis (blue staining) in regenerated area at 21 dpr (J-L).


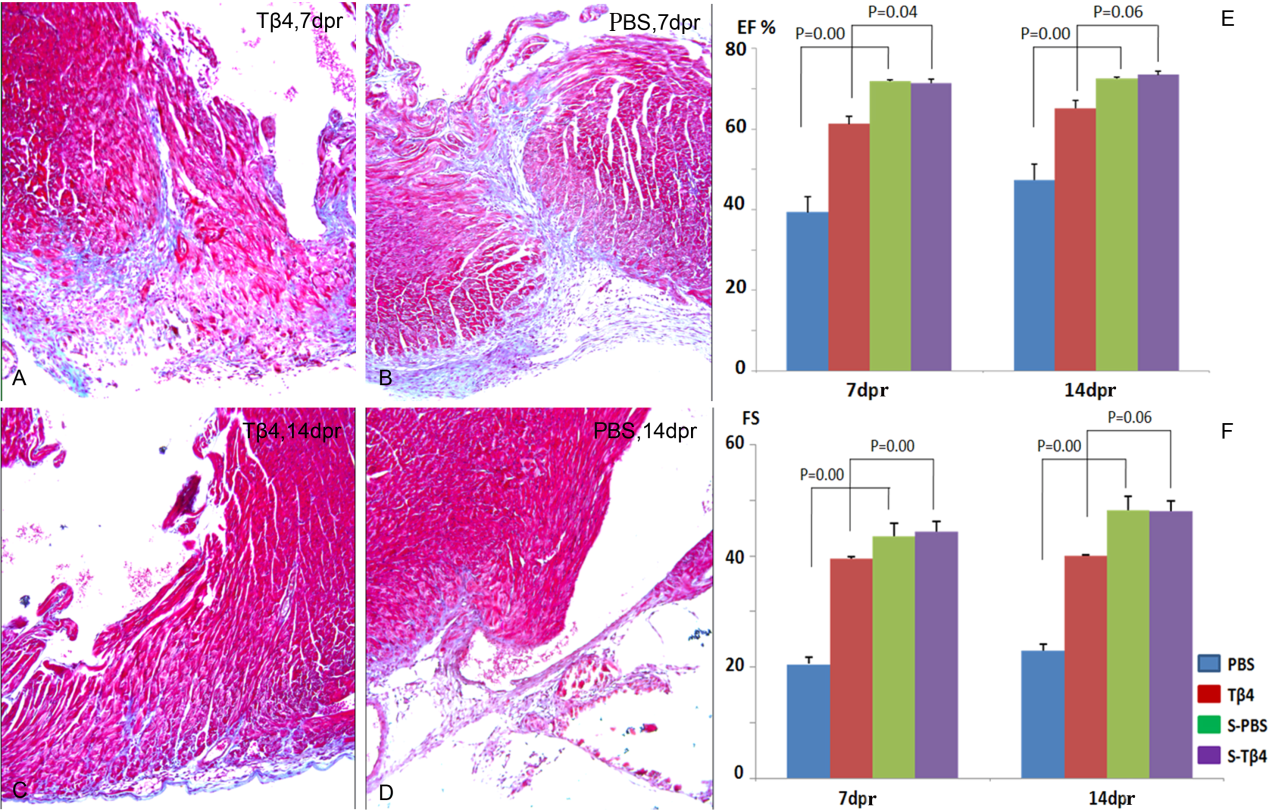


**Supplementary figure 2: Heart function and masson staining at 7 dpr and 14 dpr.** At 7dpr in Tβ4- and PBS-treated group hearts, the deposition of epicardial extracellular matrix was observed in the injury region(A,B). At 14dpr, most of lost myocardium were regenerated with minimal evidence of cardiac fibrosis in Tβ4- treated group heart(C), while obvious cardiac fibrosis and remodeling myocardium could be found in injury region in PBS-treated group heart(D).At 7dpr, the EF and FS Tβ4-treated group were significant lower than that of sham operated group of both Tβ4-treated (STβ4- treated), while at 14dpr, there was no dramatic difference of the EF and FS between Tβ4-treated and STβ4- treated group（E,F）.


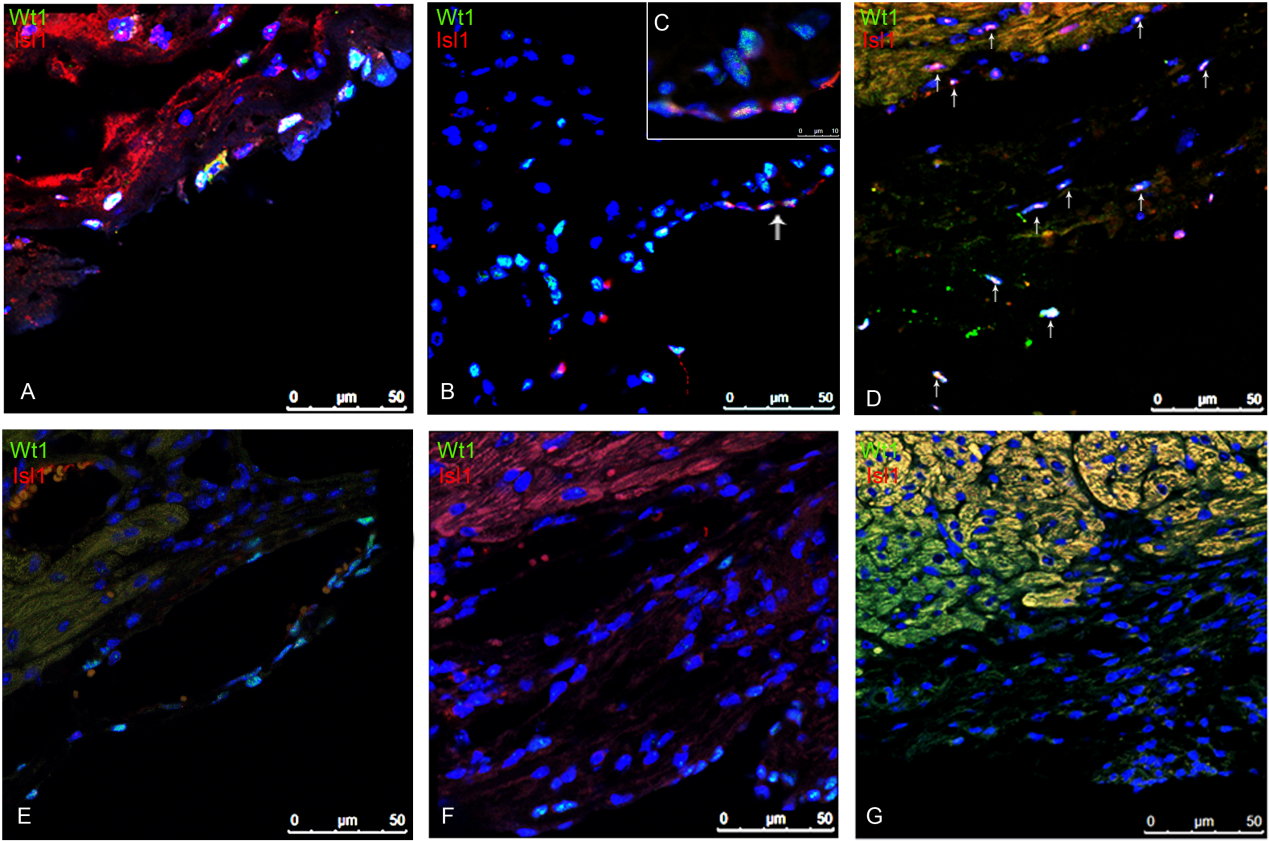


**Supplementary figure 3: Co-localization of Wt1 with Isl1 in the heart regeneration process of 7-day-old mice.** Immunohistochemistry exam showed that in Tβ4-treated mice heart some Wt1^+^ EPDCs were positive for Isl1, indicated by white arrow, at 2 dpr (A) and 7 dpr (B), the inset was a high-magnification image of Wt1^+^ EPDCs positively stained for Isl1 (C). At 14 dpr, white arrows indicated these Wt1^+^/Isl1^+^ cells migrated into sub-epicardial areas and the myocardial areas adjacent to injury area (D). But no Isl1 expression was observed in the hearts of PBS control mice at 2 dpr (E), 7 dpr (F) and 14 dpr (G).


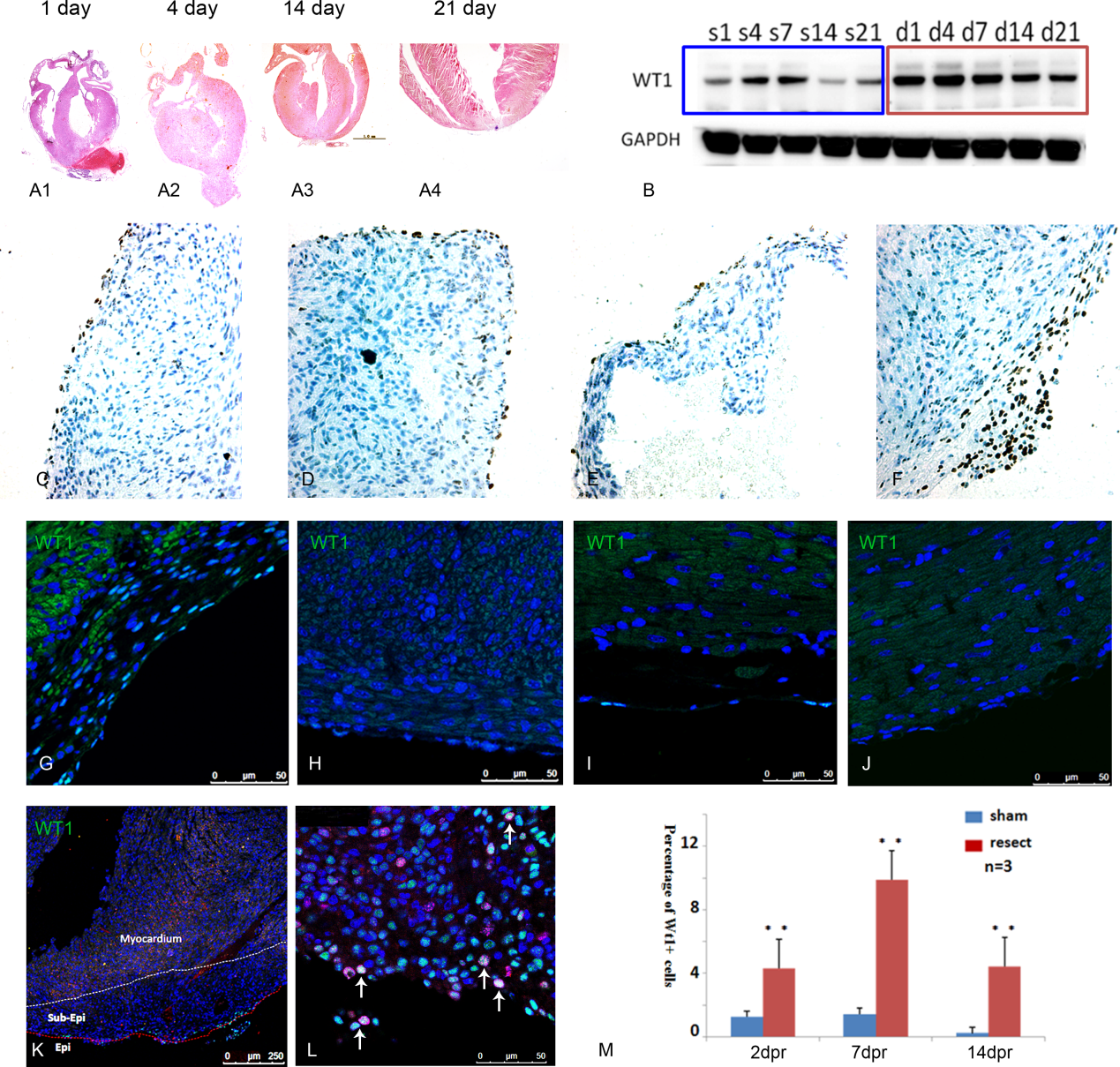


**Supplementary figure 4: Activation of epicardium after apical resection in 1-day-old mice.** Hemat oxylin and eosin (H&E) staining of the neonatal 1-day-old mouse heart at 1, 4, 14, and 21 dpr show regeneration process of the ventricular myocardium(A1-4). Western blotting indicated Wt1 was stronger expressed in resected heart than in sham operation heart(B). Wt1 was expressed in the epicardium covering ventricles(C), atria(D), and outflow tract(E), pronounced in the vicinity of injury area within the first 7 days. Epicardium marked by Wt1 increased from one layer to several layers(G) and enveloped the wound with Wt1^+^ EPDCs migrated into subepicardial area(K). At 21 dpr, only few Wt1^+^ cell could be observed(I). For sham-operated mice, A marginal level of Wt1 expression was observed in epicardial cells at 4 and 21 dpr(H,J). White arrows indicated Ki67^+^/Wt1^+^ double positive cells, which exhibited that most Wt1^+^ cells were in mitosis stage(L).Quantification of Wt1^+^ cells by HCS showed that the percentage of Wt1^+^ cells in the entire heart was significantly higher than that in the sham-operated hearts at 2,7 and 14 dpr (M). (n=3-6 each group, Mean±s.d.). White dashed line indicated resection plane; red dashed line represented the border between epicardial and sub-epicardial areas. S1,S4,S7,S14,S21means 1,4,7,14,21 days after sham operation and d1,d4,d7,d14,d21 means 1,4,7,14,21 dpr.


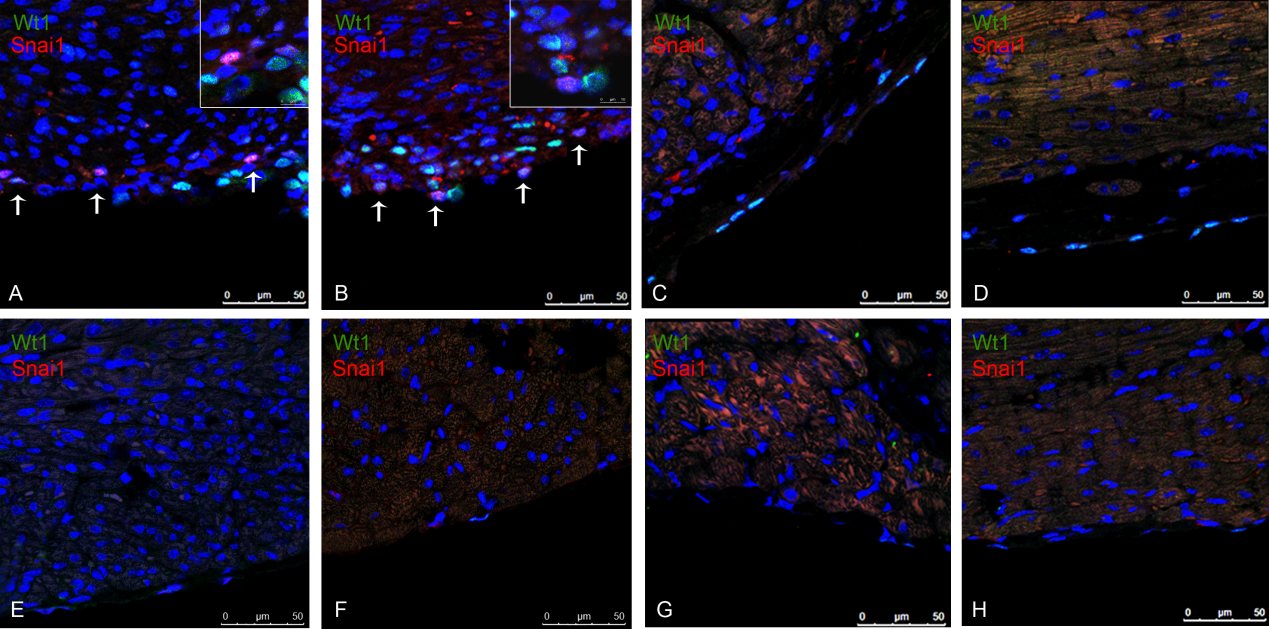


**Supplementary figure 5: The expression patern of snai1 in the heart regeneration process of 1-day-old mice.** By immunohistochemical staining, Snai1^+^/Wt1^+^ double positive cells were seen in the epicardial and sub-epicardial regions at 2 and 4 dpr(A,B), Which were indicated by white arrows and the insets were the high-magnification images of Snai1^+^/Wt1^+^ double positive cells. At 14 and 21 dpr, almost no Snai1^+^ cell could be seen,(C,D) . For sham-operation group, no Snai1 expression was found at 2, 4, 14 and 21 dpr (E-H).


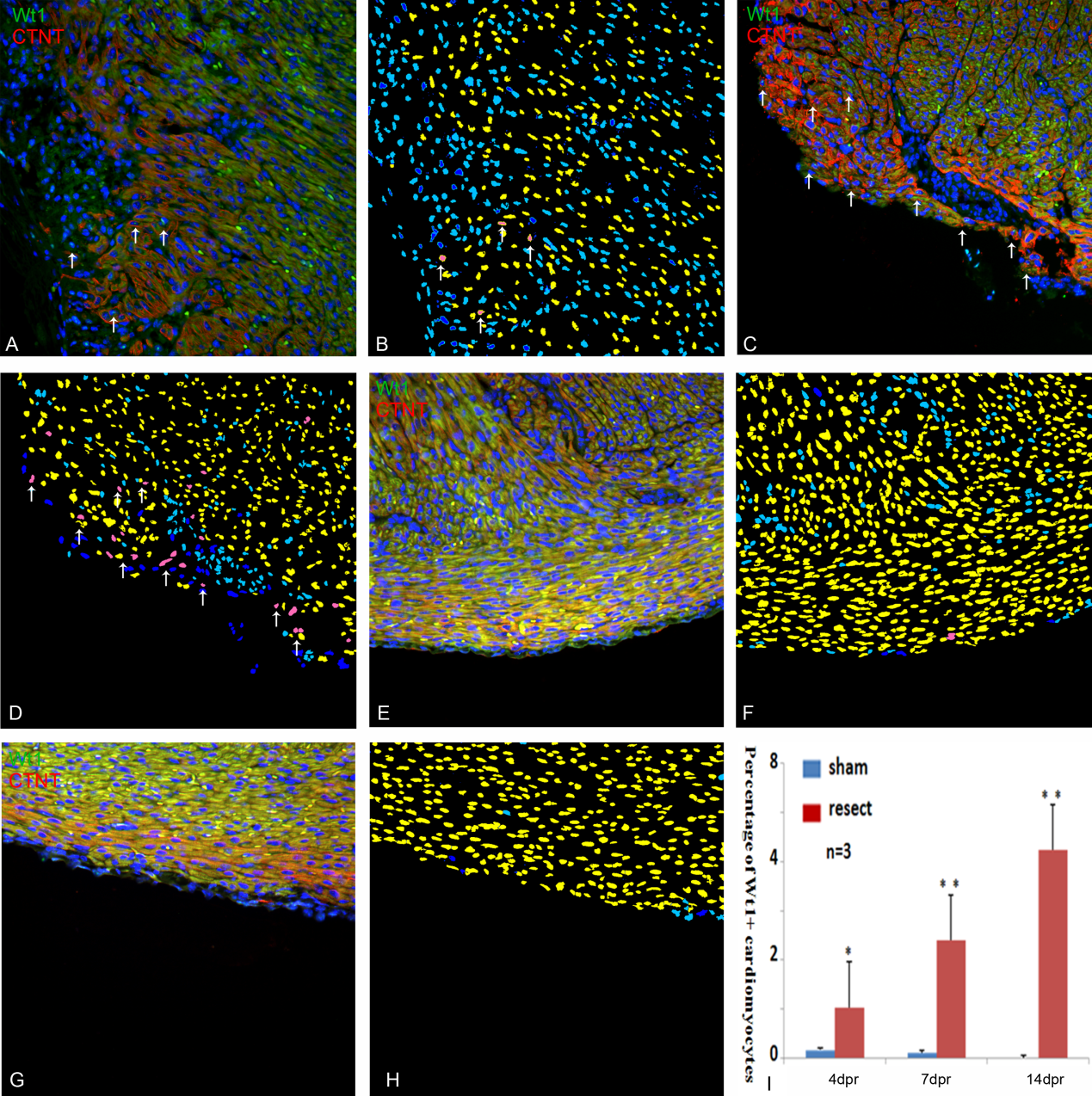


**Supplementary figure 6: Microscopy images and image reconstruction for Wt1 and cTNT staining in 1-day-old mice heart.** Using HCS, a series of Wt1 and cTNT staining heart slices were scanned at 4, 7, 14 dpr in both sham and resected mice heart. There are 2 slicing through every heart and 3-4 mice heart in each group at every time point. All 6-8 slides in each group at every time point were screened and imaged at high speed. Four microscopy images and four image reconstructions were showed in this figure. In the microscopy image at 7dpr, Wt1^+^/cTNT^+^ double cells were indicated by white arrows(A), which were signified by red points in corresponding image reconstruction, also indicated by white arrows(B). At the microscopy image of 14 dpr, white arrows indicated plenty of Wt1^+^/cTNT^+^ cells in injury region (C), in corresponding image reconstruction of which the red points, signifying the Wt1^+^/cTNT^+^ cells, indicated by white arrows (D). For sham-operation group, at 7 and 14dpr, no obvious Wt1^+^/cTNT^+^ cells were found in the microscopy image(E,G) and no red points were seen in image reconstructions(F,H). The percentage of Wt1^+^/cTNT^+^ cells of each slicing were analyzed by intelligent analysis software and the results showed that in resected group the percentage of these cells in the entire heart was higher than that in sham group at 4, 7 and 14 dpr(I).


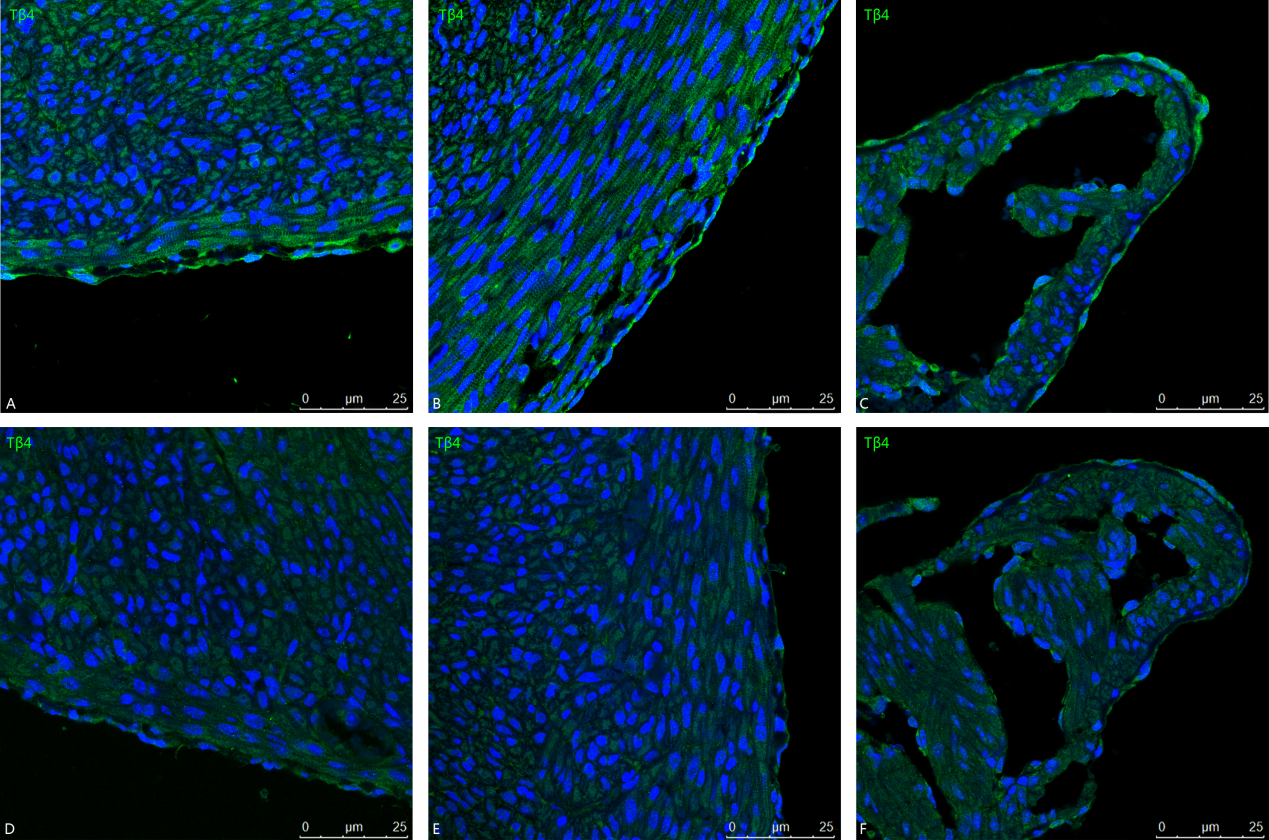


**Supplementary figure 7:** **Intraperitoneal injection of Tβ4 could elevate the cardiac exogenous Tβ4 level in heart of postnatal mice.** After treating 1-day-old neonates by Tβ4 or PBS for 7 days, the expression level of Tβ4 was detected by immunofluorescence technique in both groups. There were robust Tβ4 signal in the epicardial and sub-epicardial regions(A-C) , While in PBS control group, only sparse Tβ4 signal could be seen(D-F).

**Supplementary table 1: The percentage of Wt1^+^ cells in whole heart in the heart regeneration process of 1-day-old mice heart.**

| The percentage of Wt1^+^ cells at every time point | Rescection group | Sham group | P-value |
| --- | --- | --- | --- |
| 2dpr | 4.3±0.8% | 1.4±0.4% | 0.006 |
| 7dpr | 9.9±2.5% | 1.2±0.5% | 0.004 |
| 14dpr | 4.4±0.5% | 0.2±0.1% | 0.008 |

**Supplementary table 2: The percentage of Wt1^+^/cTNT cells in whole heart in the heart regeneration process of 1-day-old mice heart.**

| The percentage of Wt1^+^/cTNT^+^ cells at every time point | Rescection group | Sham group | P-value |
| --- | --- | --- | --- |
| 4dpr | 1.032±0.216% | 0.171±0.038% | 0.013 |
| 7dpr | 2.396±0.277% | 0.117±0.024% | 0.002 |
| 14dpr | 4.227±1.016% | 0.016±0.003% | 0.002 |
